# A Novel Phenylpyrrolidine Derivative: Synthesis and Effect on Cognitive Functions in Rats with Experimental Ishemic Stroke

**DOI:** 10.3390/molecules26206124

**Published:** 2021-10-11

**Authors:** Denis A. Borozdenko, Aiarpi A. Ezdoglian, Tatiana A. Shmigol, Darya I. Gonchar, Dmitri N. Lyakhmun, Dmitri V. Tarasenko, Yaroslav V. Golubev, Elvira A. Cherkashova, Daria D. Namestnikova, Ilya L. Gubskiy, Alexey A. Lagunin, Leonid V. Gubsky, Vladimir P. Chekhonin, Sophia S. Borisevich, Maxim A. Gureev, Anastasia D. Shagina, Nina M. Kiseleva, Vadim V. Negrebetsky, Yuri I. Baukov

**Affiliations:** 1 Department of Medicinal Chemistry and Toxicology, Pirogov Russian National Research Medical University, 117997 Moscow, Russia; borozdenko@phystech.edu (D.A.B.); forsmthgood@yandex.ru (A.A.E.); tatishtish@gmail.com (T.A.S.); daria.gonchar.1999@mail.ru (D.I.G.); lyahmun@mail.ru (D.N.L.); tarasdima0320@gmail.com (D.V.T.); yagolubev@gmail.com (Y.V.G.); alagunin@rambler.ru (A.A.L.); gubskii@mail.ru (L.V.G.); nasya.shagina@gmail.com (A.D.S.); kiseleva.67@mail.ru (N.M.K.); nmr_rsmu@yahoo.com (V.V.N.); 2 Department of Neurology, Neurosurgery and Medical Genetics, Faculty of Medicine Federal State Budgetary Institution, Federal Center of Brain Research and Neurotechnologies, Federal Medical Bio-logical Agency, 117997 Moscow, Russia; tchere@yandex.ru (E.A.C.); dadnam89@gmail.com (D.D.N.); gubskiy.ilya@gmail.com (I.L.G.); 3 Institute of Biomedical Chemistry, 119121 Moscow, Russia; 4 Department of Medical Nanobiotechnologies, Pirogov Russian National Research Medical University, 117997 Moscow, Russia; chekhoninnew@yandex.ru; 5 Laboratory of Physical Chemistry, Ufa Institute of Chemistry UFRS RAS, pr. Oktyabrya 71, 450054 Ufa, Russia; monrel@yandex.ru; 6 Laboratory of Bioinformatics, Research Center “Digital Biodesign and Personalized Healthcare”, I.M. Sechenov University, 119991 Moscow, Russia; max_technik@mail.ru; 7 Laboratory of Bioinformatics and Computational Modelling of Biological Systems, Department of Computational Biology, Sirius University of Science and Technology, 354340 Sochi, Russia

**Keywords:** synthesis, neuroprotective activity, transient MCAO, experimental ischemic stroke, behavioral tests

## Abstract

We performed an in silico, in vitro, and in vivo assessment of a potassium 2-[2-(2-oxo-4-phenylpyrrolidin-1-yl) acetamido]ethanesulfonate (compound **1**) as a potential prodrug for cognitive function improvement in ischemic brain injury. Using in silico methods, we predicted the pharmacological efficacy and possible safety in rat models. In addition, in silico data showed neuroprotective features of compound **1**, which were further supported by in vitro experiments in a glutamate excitotoxicity-induced model in newborn rat cortical neuron cultures. Next, we checked whether compound **1** is capable of crossing the blood–brain barrier in intact and ischemic animals. Compound **1** improved animal behavior both in intact and ischemic rats and, even though the concentration in intact brains was low, we still observed a significant anxiety reduction and activity escalation. We used molecular docking and molecular dynamics to support our hypothesis that compound **1** could affect the AMPA receptor function. In a rat model of acute focal cerebral ischemia, we studied the effects of compound **1** on the behavior and neurological deficit. An in vivo experiment demonstrated that compound **1** significantly reduced the neurological deficit and improved neurological symptom regression, exploratory behavior, and anxiety. Thus, here, for the first time, we show that compound **1** can be considered as an agent for restoring cognitive functions.

## 1. Introduction

Neurological disorders are one of the leading sources of premature mortality, ranking second after cardiovascular diseases, and have become the major problem in terms of the percentage of temporary or permanent disabilities among survivors [1]. The number of patients suffering from these conditions is growing, along with the aging of the population.

Among all neurological disorders, post-ischemic stroke plays a huge role in the disability level and significantly decreases the quality of life of the patients who have survived a stroke. Blood flow restoration leads to reperfusion trauma and blood–brain barrier damage. Infarct zone formation is associated with a local and systemic immune response, including brain edema, proinflammatory cytokine activation, and brain cell apoptosis [2]. All of these mechanisms are considered potential targets for new therapy. Despite all attempts, reperfusion therapy, intravenous thrombolysis with a tissue plasminogen activator, and endovascular mechanical thrombectomy are the only available efficacious treatments [3]. However, these methods are not safe and have a very limited time for administration. Unfortunately, during the COVID-19 pandemic, many neurological effects of the viral infection were also described, therefore dramatically increasing the importance of the identification of new therapeutic agents for cognitive and memory dysfunction, not only in post-ischemic stroke management.

Within the past decade, several new drugs for treating cognitive and memory dysfunction after ischemic stroke have been proposed. Among them, of particular interest are agents containing the pyrrolidine-2-one fragment as a pharmacophore, belonging to the racetam family, which are currently the agents of choice for cognitive deficit treatment and memory improvement [4].

The biological activity of these compounds is strongly dependent on conformational changes in the chiral center and on the introduced pharmacophore groups [5]. Taking that into consideration, the drugs from the racetam family, which differ from each other in the structure of the substituents around the pyrrolidine-2-one heterocycle, have maintained a well-deserved reputation as leading therapeutic agents for the improvement of cognitive functions, attention abilities, and mental conditions associated with head traumas, stroke, aging, and age-related pathologies and seizures [4]. However, the majority of known racetam family members are not water-soluble, which introduces limitations on intravenous administration. Previously, our group proposed that the “silyl” method of *N*-alkylation of lactams is based on the condensation of their *N*-trimethylsilyl derivatives and organohalides with the formation of *N*-substituted lactams and the corresponding trimethylhalogenosilanes [6]. There are still many unanswered questions about the efficacy safety and biological properties of the racetam family members.

We hypothesized that the potential therapeutic features of racetam family members are not fully understood and there could be members with better efficacy and safety features than available options. To identify new therapeutic agents for neurodegenerative diseases, an in silico assessment of functional derivatives of the racetam family was conducted and the lead candidate was characterized using molecular docking. We further investigated blood–brain permeability using in silico and in vivo models of middle cerebral artery occlusion (MCAO). Afterward we studied the neuroprotective activity of the identified compound in intact and MCAO rats by looking at the activity and behavioral patterns of the animals, and accessing the neurological deficit. Along with the behavior test, we also conducted a cerebral MRI and measured the tendency of cerebral damage. Therefore, for the first time, we present a new racetam family member as a potential therapeutic agent for neurological disorders.

## 2. Results and Discussion

### 2.1. Chemistry

**Scheme 1:**
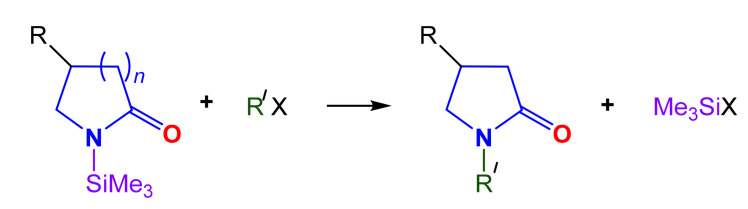
“Silyl” method of *N*-alkylation of lactams; *n* = 1, R = H, Ph; *n* = 2,3, R = H; X = Cl, Br; R’X = ClCH_2_Ph, BrCH_2_Ph, ClCH_2_COOR” (R” = Et, *i*-Am, (CH_2_COOEt), BrCH_2_COOR” (R” = Me, Et, *i*-Am, CH_2_COOEt, SiMe_3_), ClCH_2_CONEt_2_).

Previously, we proposed the “silyl” method of the *N*-alkylation of lactams based on the condensation of their *N*-trimethylsilyl derivatives and organohalogenides with the formation of *N*-substituted lactams and the corresponding trimethylhalogenosilanes [6] (Scheme 1). Using this method, several topological analogs of phenotropil were obtained, including lactam-containing peptides [7].

**Scheme 2:**
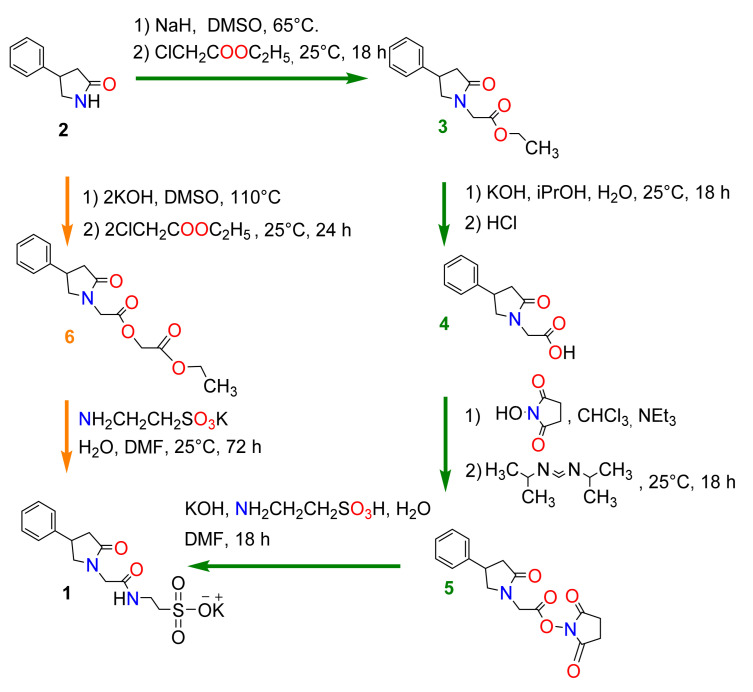
Representative synthetic routes for compound **1**.

Here, we synthesized compound **1** by two different methods (Scheme 2).

The first laboratory’s method (**2**→**6**→**1**, Scheme 2, method 1) is the synthesis of activated ester **6** by the alkylation of the potassium salt obtained by keeping the starting lactam **2** in a solution of potassium hydroxide in DMSO for 4 h at a temperature of 110 °C. The alkylation was carried out with a twofold excess of ethyl ester of chloroacetic acid. The activated ester **6** was isolated by vacuum distillation with a yield of 65%. The target compound **1** was obtained by adding the potassium salt of taurine to the compound **6** with a yield of 97%.

Due to the fact that this method had a low compound **6** yield after vacuum distillation (due to contamination with thermal decomposition products), we also designed a different four-step approach (**2**→**3**→**4**→**5**→**1**, Scheme 2, method 2). The alkylation of 4-phenylpyrrolidinone-2 (**2**) was performed using ethyl chloroacetate in the presence of sodium hydride. The product of this step ethyl 2-(2-oxo-4-phenylpyrrolidin-1-yl)acetate (**3**) was isolated by column chromatography with a nearly quantitative yield (98%).

Alkaline hydrolysis of ester **3** was performed in the presence of potassium hydroxide in a mixture of water and isopropanol. Upon further extraction, 2-(2-oxo-4-phenylpyrrolidin-1-yl)acetic acid (**4**) was obtained with a yield of 87%.

Acid esterification compound **4** was performed with *N*,*N*-hydroxysuccinimide in the presence of *N,N*-diisopropylcarbodiimide. The extraction and subsequent treatment with water produced 2,5-dioxopyrrolidin-1-yl 2-(2-oxo-4-phenylpyrrolidin-1-yl)acetate (**5**) with a yield of 94%. At the final stage, the activated ester **5** was treated with a solution of taurine in potassium hydroxide, producing the target potassium 2-[2-(2-oxo-4-phenylpyrrolidin-1-yl)acetamido]ethane-1-sulfonate (**1**) as a beige crystalline solid with a yield of 94%. Thus, this method can be used for industrial production.

### 2.2. The Structure-Activity Relationship (SAR)

The PASS Online web service [7,8] was used to predict possible pharmacotherapeutic effects and mechanisms of the action of synthesized compound **1**. A relationship between the structure and activity of *N*-substituted lactam derivatives and neuroprotective activity was revealed. After the introduction of taurine salt into the structure, the antihypoxic activity rose. In addition, a high probability of antianginal, nootropic, antihypoxic, and cytoprotective effects for compound **1** was predicted (Table 1). These results are supported by previously shown data for other racetam family members [4]. One of the expected mechanisms of action is the “neurotransmitter antagonist”, which is related to a nootropic effect (Table 1). The results of the prediction of nootropic/cerebral anti-ischemic effects and related mechanisms of action highlighted the rationale for further experimental investigation.

Before initiating in vitro and in vivo experiments, we also calculated predicted LD_50_ values for rats using the GUSAR Online Acute Toxicity web service. According to the prediction, compound **1** has a low toxicity score and is safe if administered intravenously and intraperitoneally. Considering the predicted safety and mechanisms of action, we investigated compound **1** in in vitro and in vivo experiments.

### 2.3. In Vitro Studies

**Figure 1:**
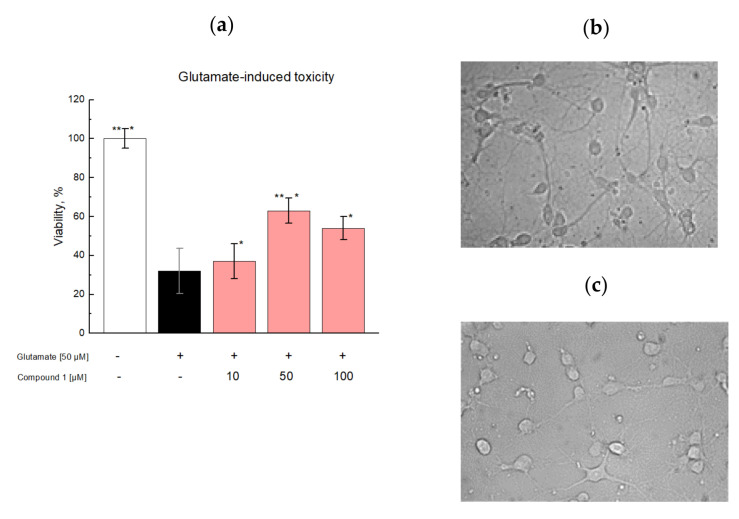
(**a**)—compound **1** effects on glutamate-mediated neurotoxicity in a primary newborn rat cortical neuron culture. Newborn Wistar rat cortical neurons were cultured with glutamate for 24 h and afterward treated with 10 μM, 50 μM, 100 µM of compound **1**; after 24 h, 3(4,5-dimethylthiazol-2-yl)-2.5-diphenyltetrazolium bromide (MTT) assay was used to assess the cell viability. * A significant difference compared with the glutamate-free control, *P* < 0.05. ** A significant difference compared with the glutamate control, *P* < 0.05. The results are expressed as the mean ± standard error of the mean for three independent experiments (*n* = 8). The data were normally distributed according to Kolmogorov–Smirnov test. One-way ANOVA was used; (**b**)—cortical neurons before glutamate treatment; (**c**)—cortical neurons after treatment with glutamate 24 h.

To confirm the predicted biological activity, we adopted a glutamate-induced excitotoxicity in the rat cortical neurons model. This work was based on previous findings that glutamate is a well-known excitatory CNS neurotransmitter, which contributes to normal neural transmission, development, differentiation, and plasticity [9]. However, excessive extracellular glutamate levels cause uncontrolled continuous neuron depolarization, which can lead to neuronal death. Excitotoxicity is associated with several neurodegenerative conditions, including Huntington’s disease, Alzheimer’s disease, amyotrophic lateral sclerosis, Parkinson’s disease, and stroke/traumatic brain injury [10]. To see whether compound **1** has neuroprotective effects, we studied the influence of compound **1** in glutamate-treated cortical neurons isolated from newborn Wistar rats (0–1 day). Compound **1** showed a significant neuroprotective effect against glutamates, compared to the control group. The maximum protective effect was observed at 50 µM of compound **1**; moreover, the cell survival rate increased by 37% after exposure to 50 µM glutamate (Figure 1).

It should be noted that the neuroprotective effect of compound 1 decreases at a dose of 100 μM. It was shown by X-ray crystallography that some racetam family members are presented as two independent enantiomer molecules, stabilized by intermolecular hydrogen bonds N–H···O [6]. As compound **1** and these members have a lot in common, we believe that compound **1** also forms aggregates, which may cause an observed efficacy reduction.

### 2.4. In Vivo Studies

**Scheme 3:**
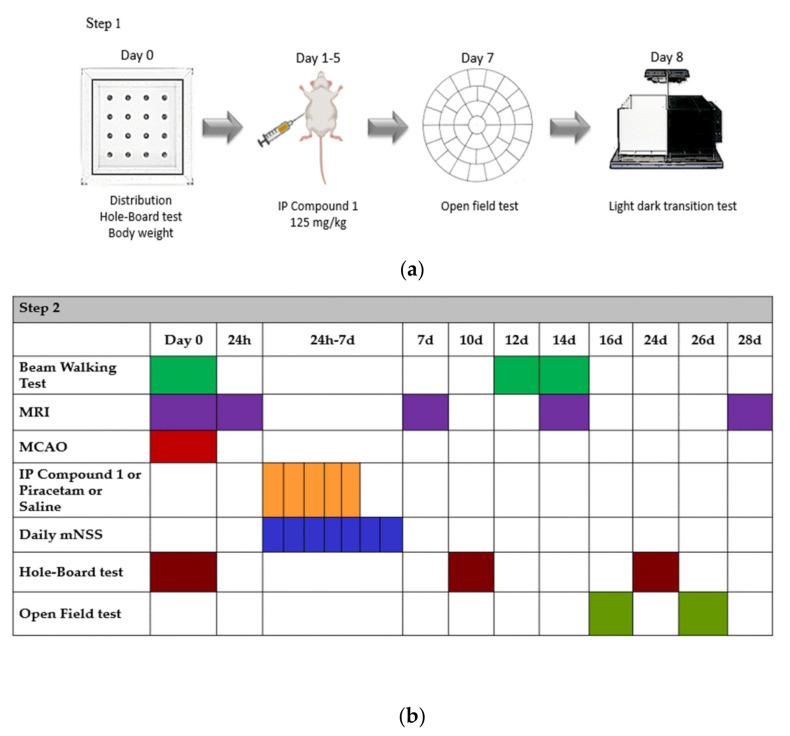
Study design. (**a**)—Step 1: Investigation of the effects of compound **1** on the behavior of intact animals; (**b**)—Step 2: Investigation of the effects of compound **1** on the treatment of animals with MCAO.

The neuroprotective activity of compound **1** predicted in molecular docking studies and supported by in vitro tests was evaluated in vivo using a transient MCAO model. The study consisted of two steps: Step 1 investigated the effect of compound **1** on the behavior of intact animals, while Step 2 assessed the effect of compound **1** on animals with MCAO (Scheme 3).

#### 2.4.1. Behavioral Effects of Compound **1** in Intact Animals

Neurotropic effects of compounds are necessary for the treatment of neurodegenerative disorders and play a huge role in memory dysfunction management. Here we compared the effects of compound **1** with piracetam as a positive control and used normal saline as a negative control [4]. Patients with cerebral ischemia-induced short-term memory/cognitive deterioration benefited from piracetam. It was shown that around 25% of the patients improved their memory and cognitive functions after piracetam; therefore, we selected piracetam as a known effective racetam family member.

Before the study, the intact animals were subjected to the hole-board test (HBT), weighed, and stratified into three uniform groups in terms of orientational/exploratory activity and weight. Compound **1**—125 mg kg^−1^ b.w., piracetam—300 mg kg^−1^ b.w. and normal saline—2 mg kg^−1^ b.w. were administered intraperitoneally for 5 days. On day 7, we performed OFT (open field test), followed by LDB (light—dark box test) on day 8 (Scheme 3, Step 1). These tests assess the activity and describe the behavior of the animals. According to OFT, compound **1** decreased the vertical activity and the immobility time compared to the saline-treated group (Table 2).

The LDT showed a decreased anxiety and increased exploratory activity compared to the negative control group (Table 3). There was no significant difference observed compared to the piracetam group. According to these results, compound **1** has better neurotropic activity than piracetam, as it reduces the immobility time and improves the number of center sector crossings and vertical activity (Table 2). In addition, compound **1** reduced the time in the light compartment (Table 3). Thus, compound **1** increases the activity and curiosity of animals and reduces the anxiety levels. It was shown that these effects on the behavior may lead to cognitive function improvement and decrease the severity of depression [11].

#### 2.4.2. Effects of Compound **1** in Rats with MCAO Model

A MCAO animal model was used to characterize the effects of compound **1** on the development and resolution of stroke. At the baseline, we performed the MRI and hole-board test before MCAO surgery. Once animals recovered after the surgery, we stratified them based on their background psycho-emotional status and performed a control MRI to access whether the occlusion was sufficient or not. From 24 h to day 5, compound **1**, piracetam, or normal saline were administered intraperitoneally. From 24 h to day 7, the neurological status was assessed daily according to the neurological scale (mNSS). By the end of day 7, animals underwent MRI. In addition, MRI was performed on days 14 and 28 (Shceme 3)The hole-board test was repeated on days 10 and 24. The degree of motor function impairment, the deficit of exploratory behavior, and the recovery process were assessed in the beam walking test (BWT), which was performed twice on days 12 and 14. An open field test was carried out on days 16 and 26.

#### Survival Rate

**Figure 2:**
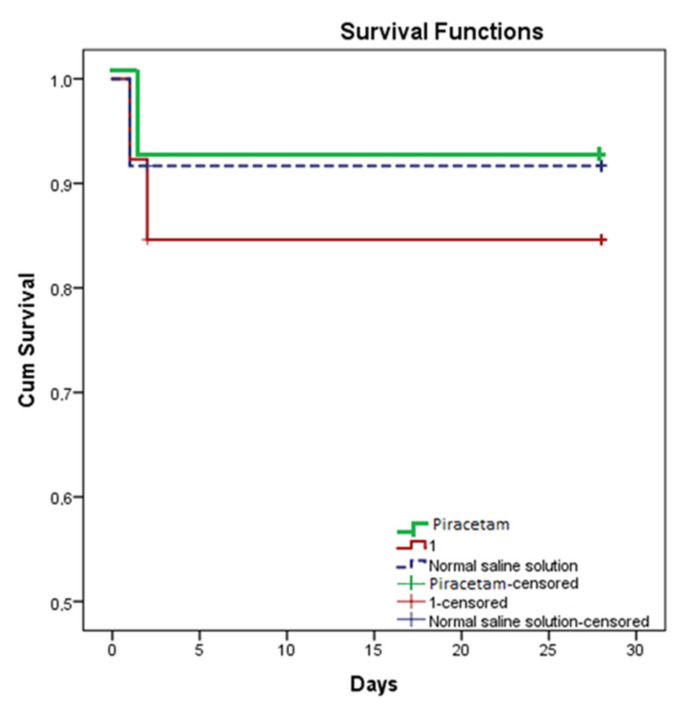
Kaplan–Meier survival curves for rats of the group with compound **1** administration (red line), piracetam (green line), and normal saline solution (blue line) within 28 days of observation. No significant differences between groups of animals were found.

Survival rates were estimated within 28 days after the first administration of compound **1**, piracetam, or saline. Kaplan–Meier survival curves are presented in Figure 2. The comparison of experimental groups was made by the log-rank test and showed no significant differences in the survival of animals.

In each group, there was one death case on day 1 after MCAO and one animal per group reached a humane endpoint on day two. One animal from the compound **1** group was humanely killed on day two after MCAO. Thus, there was no significant difference observed in viability and all deaths were not associated with administered compounds.

#### Cerebral Infarct Volume

**Figure 3:**
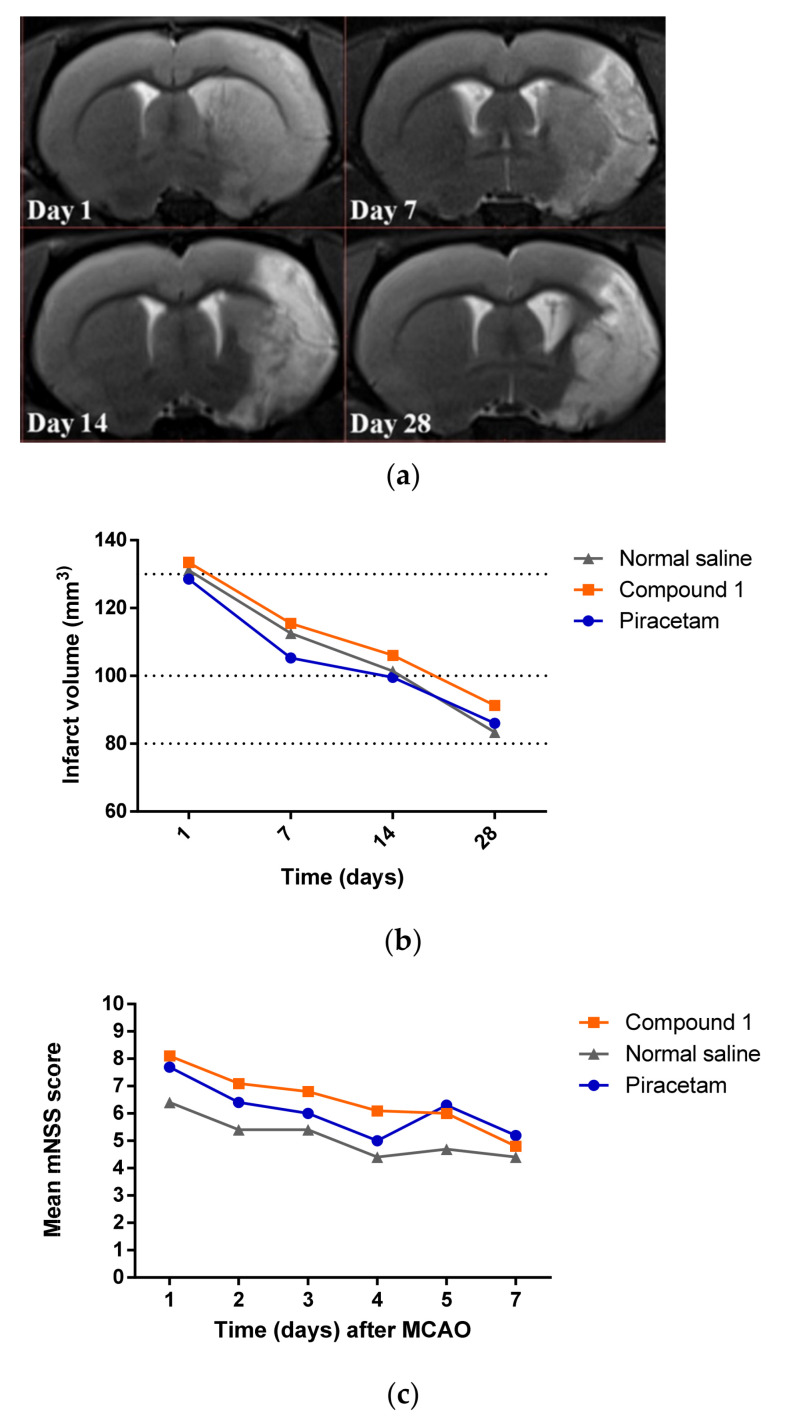
(**a**)—T2-weighted MRI images of the rat brain on postoperative days 1, 7, 14, and 28. The hyperintense zones correspond to ischemic lesions; (**b**)—infarct volume in mm^3^ at different time points after MCAO (24 h) and treatment initiation (days 7, 14, 28); (**c**)—mean mNSS score after MCAO in rats treated with compound **1**, piracetam, and normal saline. There were no statistically significant differences observed between the three groups.

Along with the behavior test, we also conducted cerebral MRI to study the influence of compound **1** compared to piracetam; here, normal saline was used as a negative control. No significant difference in the infarct volume at 24 h was observed between the groups before treatment (compound **1**: 133.6 ± 24.1 mm^3^, saline group: 131.1 ± 28.2 mm^3^, piracetam: 128.5 ± 15.5 mm^3^). The infarct volume had a similar reduction rate for all three groups on days 7, 14, and 28. By day 28, the infarct volume was 91.2 ± 18.9 mm^3^ for compound **1**; 83.1 ± 15.6 mm^3^ for saline, and 86.0 ± 13.5 mm^3^ for piracetam, p = 0.17. Examples of MRI images are shown in Figure 3.

#### Neurological Deficit

Along with tracking changes in the volume of the brain lesion, the effect of the compounds on the improvement of the neurological deficit was studied. The mNSS scale was used to assess the severity of the neurological impairment. It includes a complex of motor (muscle condition and abnormal movements), sensory (visual, tactile, and proprioceptive), reflex, and balance tests [12].

The analysis of the neurological deficit showed that the regression of neurological symptoms occurred more quickly in the groups treated with compound **1** and piracetam than in the saline group, and accounted for 35% in the compound **1** group and 25% in the piracetam group. Rats had impaired motor function on the contralateral side to the MCAO lesion, as well as impaired coordination. The vestibular function recovered before motor function in the compound **1** and piracetam groups, whereas, in the saline group, the neurological deficit improved stepwise. However, no significant differences in the extent of the neurological damage were found between the groups when comparing the neurological scale data on day 7 after stroke. This is consistent with other studies showing a high recovery of neurological functions over time [13].

**Figure 4:**
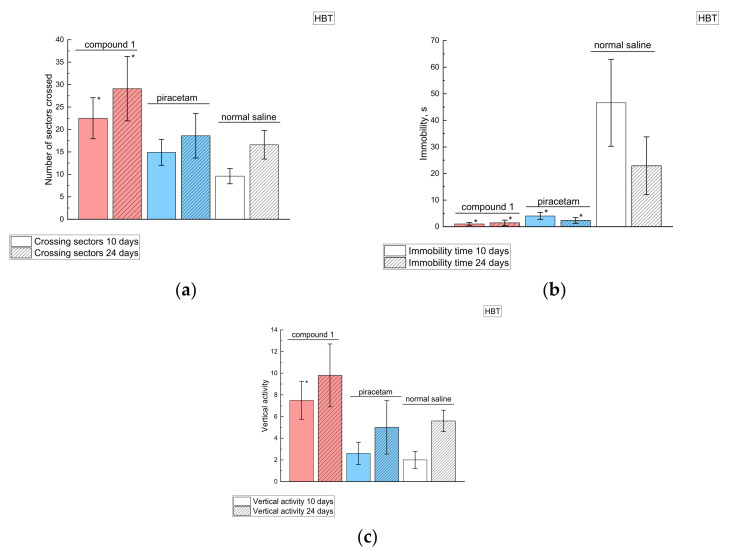
Results of the HBT test. (**a**)—the average number of sectors crossed; (**b**)—immobility time; (**c**)—vertical activity. Empty rectangle—data for day 10; a rectangle with shading—data for day 24. The results are expressed as the mean ± standard error (SD). * *p* < 0.05. Animals in the experimental group demonstrated increased motor activities and decreased anxiety.

The tentative exploratory behavior of the animals was tested in a hole-board and an open field test. The HBT test was carried out on days 10 and 24 after MCAO, and OFT on days 16 and 26. We alternated two tests with different arena areas and shapes to reduce the possible memorization by animals and more objectively assess the motor activity and anxiety of the rats. Compound **1** significantly increased the total locomotor activity of the animals (Figure 4a) and decreased the freezing time (Figure 4b) on days 10 and 24. In addition, on day 1 in the compound **1** treated group, we observed an increase in orientational activity (Figure 4c).

On days 10 and 24 in the burrow chamber test, rats from the compound **1** group showed a statistically significant increase in total locomotor activity (Figure 4a), a decrease in freezing time (Figure 4b), and an escalation of orientational activity on day 10 (Figure 4c).

**Figure 5:**
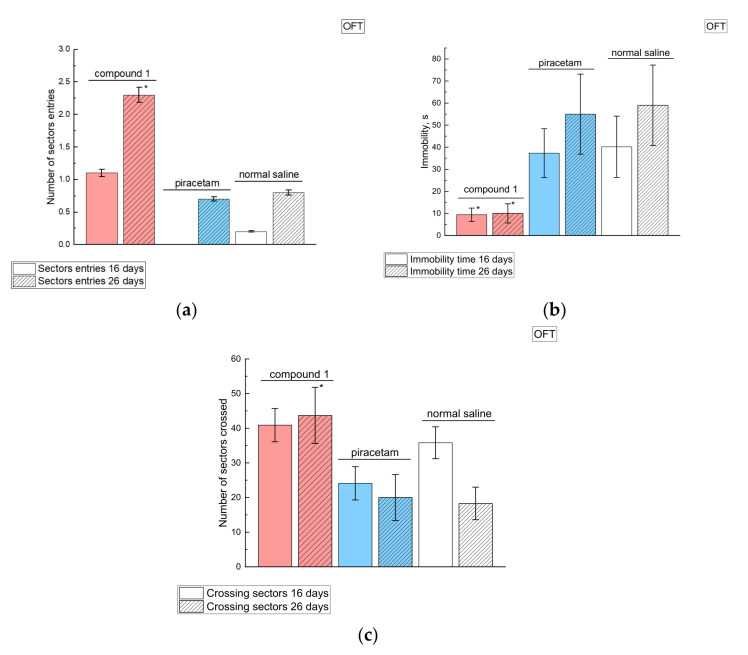
OFT test. (**a**)—the average number of crossed central sectors; (**b**)—immobility time; (**c**)—the average number of sectors crossed. Empty rectangle—data for day 16; a rectangle with shading—data for 26 days. The results are expressed as the mean ± standard error (SE). * *p* < 0.05.

Compound **1** was also beneficial for animals in OFT: on day 16, there was a statistically significant increase in the number of crossed areas (Figure 5a), and the immobility time was lower compared to the saline and piracetam groups (Figure 5b). We also observed a higher locomotor activity in the group receiving compound **1** (Figure 5c).

**Figure 6:**
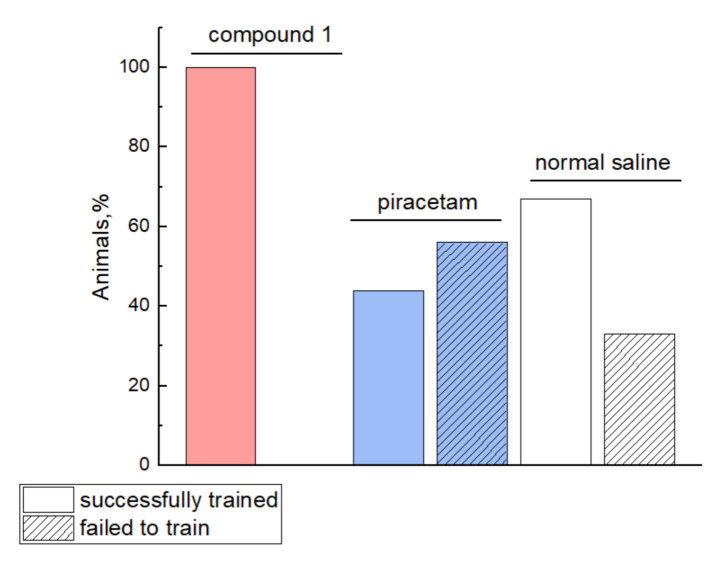
Beam walking test success rate. Animals were trained three times per day during 3 days before MCAO and on days 12–14 after MCAO. On day 14 after MCAO, the test and the number of steps, errors, and slides were counted. Solid bars represent animals who reached the black box. Stripes represent animals who fell from the beam, stood still, or turned back towards the light. No animals failed in compound **1** treated group (pink), more than half of the animals in piracetam group (blue) failed the training, and 30% of the animals failed in the normal saline group.

The increased locomotor activity, expanded orientational research activity, and reduced anxiety and depressive behavior in rats treated with compound **1** highlighted the possible benefits of the compound in post-ischemic stroke management. As stroke affects not only locomotor activity but also the cognitive behavior, we conducted an additional beam walking test. It is proven that depression and anxiety in the post-stroke period increase the risk of mortality and affect the functional recovery of patients [14,15]. There is a direct relationship between the severity of cognitive deficits, patients’ survival, and their successful social integration [16]. Conventionally, BWT is aimed at finding motor deficits on the contralateral side of the brain lesion. However, based on the test methodology, which was testing animals before surgery and on the 12th–14th days after surgery, we can indirectly conclude whether the memory trace is preserved or lost. If the animal can balance on the beam, but does not move towards a “safe”, dark shelter, or turns back, having passed half of the way, we assumed that the animal does not remember and is unable to recall that there is a shelter at the end of the beam. On the 12th–14th days after MCAO, the rats of different groups behaved differently in this test: the rats under the influence of compound **1** quickly walked into the dark compartment; some of the animals with piracetam injections were turned back to the light source, and two rats fell from the beam (Figure 6). Most of the animals from the saline group sat still and one rat fell. Since the hippocampus, which is a structure responsible for the formation of both reflex and long-term memory, is involved in the pathological process of ischemia, in rats, we hypothesized that the ability to reproduce and form a memorable trace can be also affected in our MCAO model. According to MRI data, the hippocampus was indeed affected by MCAO. Thus, compound **1** possibly helped to restore neural connections in the hippocampus as quickly as possible.

There were no significant differences in motor functions between the groups in the beam walking test (BWT) on days 12–14, which may indicate a complete recovery of subcortical structures (Table 4). These results are consistent with the neurological scale score, indicating that most experimental animals had no gait disturbances by day 7.

However, it is important to note that three animals in the saline group and five animals in the piracetam group after MACO failed to train for the BWT test. In addition, all animals from the experimental group were successfully trained. This may be due to an improved cognitive function by compound **1**.

### 2.5. The Blood–Brain Barrier Permeability of Compound **1**

The ability to penetrate through the blood–brain barrier (BBB) is crucial for drugs that affect the central nervous system. BBB is a selective physical, transport, and metabolic barrier, controlling the passage of molecules into and out of the brain. BBB prevents toxins, pathogens, or macromolecules from entering the brain [17]. It was shown that the high polarization of molecules limits their passive diffusion into the CNS [18]. As compound **1** is a polar molecule, we performed a computer simulation of its BBB permeability. In addition, we conducted in vivo experiments in intact animals and animals with MCAO.

First, using a computer stimulation, we calculated the ability of compound **1** to overcome BBB (Table 5). Changes in the LogPm (the logarithm of the membrane permeability in cm s^−1^) and membrane permeability correlate with each other. LogPm values for ampakines fall within the range of −4.42 to −4.92. Seletracetam and piracetam have the highest LogPm values and the best BBB permeability (Table 5). On the contrary, compound **1** has a low logPm value, which may state a low BBB permeability. Despite QikProp predictions being approximate, both calculated and experimental values correlate. Thus, high PMDCK values correspond with the higher membrane permeability. The lower the LogPm value, the worse the membrane permeability. These values fall within the range of −4.42 to −4.92 for the considered ampakines. According to the experimental data, seletracetam and piracetam have the highest LogPm values and a good BBB permeability. Compound **1** has a low logPm value, which may indicate a low BBB permeability.

Predicted brain/blood partition coefficient (QPlogBB) values of known oral racetam family members are within the range of [−0.97; −0.08], which, by itself, falls within the range of valid values [−3.0; 1.2]. A decrease in QPlogBB values corresponds to a decrease in the BBB permeability. Nefiracetam with a good BBB permeability has QPlogBB −0.08, and the polar compound **1** with a low QplogBB value (−1.5) does not cross the blood–brain barrier.

The calculated data are consistent with the experimental data in intact animals. The concentration of compound **1** was determined in the striatum and frontal cortex by HPLC MS. Sixty minutes after the intravenous administration of compound **1**, we anesthetized the rats with isoflurane. Afterward, we drained the blood from the heart, opened the chest, dissected the heart muscle, incised the left atrial appendage, and inserted an 18G needle into the right atrium. The pulmonary circulation was washed with 350 mL of 0.9% NaCl containing 1.0 mL of heparin. After the washing procedure, the rat was decapitated and the brain was removed and dissected. We determined the concentrations of compound **1** using an HPLC MS system. The concentration of compound **1** in plasma and the striatum differed by 650 times, and the concentration in plasma and the cortex differed by 630 times (Table 6).

The changes in BBB permeability persist up to 4 to 6 weeks after the ischemic stroke. In this regard, we investigated the passage of compound **1** through the BBB in the MCAO model.

Based on the results of the blood–brain barrier permeability research conducted, the concentration of compound **1** in the brain of intact animals was 300 ng g^−1^, and the concentration in rats with hemispheric stroke was 2.6 μg g^−1^. Thus, the BBB permeability increased almost 10-fold 24 h after MCAO (data can be found in Supplementary Material Figure S3 and Figure S4).

The observed increase in the BBB permeability of compound **1** in the ischemic stroke model (see Table 6) is consistent with the previous data [24]. Paliwal et al. (2018) also showed that the BBB permeability of piracetam increases 3.5-fold in animals with ischemic stroke [25].

### 2.6. Molecular Docking and Molecular Dynamic

Of note, it was shown that a concentration of only 0.3 μg g^–1^ of compound **1** in the brain of intact animals was sufficient for statistically significant changes in the behavior of animals.

Racetam family members probably interact with glutamate ionotropic receptors [26,27,28]. Based on BBB permeability data, along with the results of behavioral tests in animals treated with compound **1,** we used molecular docking to further investigate the possible receptor binding capacity of compound **1.**

**Figure 7:**
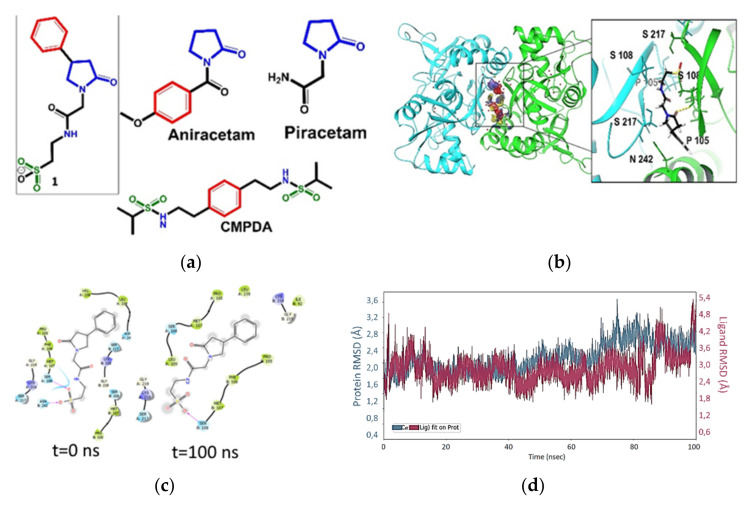
Binding of PAM of AMPA-receptor in the active site S1S2: (**a**)—comparison of structural features of the test compound **1-*R*** with aniracetam and CMPDA; (**b**)—localization of PAM binding site: donor–acceptor and hydrophobic regions are shown as blue, red, and yellow, respectively, and the location of compound **1-*R*** in the PAM binding site: water molecules are shown as red balls, hydrogen bridges as a yellow dashed line; (**c**)—results of molecular simulations for 100 ns: changes in packing of the compound **1-*R*** in the initial and final states; (**d**)—RMSD indicators of the considered system.

Molecular docking studies are based on placing a particular ligand into a receptor region, thus providing information about conformation, orientation, and organization at the receptor site [29]. Ionotropic glutamate receptors are a family of ligand-gated ion channels that are primarily localized in chemical synapses. They mediate fast excitatory neurotransmission in the mammalian central nervous system (CNS) [9]. Glutamate receptors are essential for normal cellular work, including synaptic activity and plasticity. The dysregulation of these ion channels is associated with an extensive range of neurological diseases [26]. Moreover, ischemic stroke leads to the dysregulation of AMPA receptors. Neuronal death, which is produced excitotoxicity, is induced by the excessive stimulation of neuronal glutamate receptors. Inhibiting glutamate receptors using AMPA receptor antagonists can attenuate the ischemic injury of neuronal tissue in animal experiments [27,28]. Positive allosteric modulators (PAM) improve short-term memory in humans by slowing down the deactivation of AMPA receptors [30], which makes them beneficial for the treatment of depression and other disorders and diseases of CNS [26,31,32]. The structures of known PAM of AMPA-receptors are quite diverse: racetams, a γ-lactam fragment, various benzamide type ampakines, sulfo-derivatives, etc. [33]. The test compound contains several structural descriptors found in molecules of known PAM (Figure 7a).

The presence of the γ-lactam fragment allowed us to compare compound **1** with various racetams. In particular, the sulfonic group is present in ampakine CMPDA [34], which desensitizes and deactivates the receptor in a nanomolar concentration. New derivatives of lactams can bind to the active site of PAM at the boundary of the polypeptide chains of the ligand-binding domain. The mapping of the binding site (Figure 7b) provides an estimation for the positions and sizes of the regions preferred for hydrophobic and donor–acceptor interactions.

The Glide score value for compound **1**, which characterizes the binding affinity of the compound, is comparable to the values for aniracetam and CMPDA, and over 1.5 kcal lower than the value for piracetam. It should be noted that the R-enantiomer exhibits a higher affinity for the PAM binding site. The ligand efficiency (LE) (ratio of the Glide score to the number of heavy atoms) for compound **1** is lower than those for racetams, but comparable with the values for CMPDA. The difference can be attributed to the greater number of heavy atoms in the test compound as compared to *N*-anisoyl-2-pyrrolidinone (aniracetam) and piracetam. At the same time, the IFD scores characterizing the energy of ligand–protein complexes are comparable for all studied structures.

Potential PAM bind at the LBD binding site, forming a series of non-covalent interactions (Figure 7b, Table 7).

Many of the interactions described in Table 6 are water-mediated hydrogen bridges between oxygen atoms of the lactam fragment and/or sulfonic group of compound **1-*R*** with surrounding amino acids, such as SER217, PRO105, and SER108. These amino acids can be described as functional residues because their interactions with PAM can affect the domain movements of the protein [35]. The sulfonic group of the studied compounds binds to the polar region of the active site.

The molecular simulation was performed for the most active stereoisomer compound **1-*R*** for 100 ns. For the duration of the simulation, compound **1** remains in the binding site, and no migration into the solvent is observed. However, the ligand can rotate inside the active site (Figure 7c); because of this rotation, VAL238 leaves the surface of hydrophobic contacts and is replaced with ILE92. This leads to the overall weakening of polar contacts due to the loss of interactions with ASN242 and SER217. There is also a loss of hydrogen bonding with SER108 in chain A. By the end of the simulation, ligand compound **1** becomes more accessible to the solvent. However, the ligand migration into the solvent requires more than 100 ns.

The dynamics of the RMSD indicator also highlights the receptor-binding activity of compound **1**. In Figure 7d, the red line shows the variability of the ligand position. The major rearrangement in the binding site occurs at 82–85 ns, then, the complex is stabilized for a short time, and the next rearrangement occurs at 97–98 ns. Thus, the ligand migration into the solvent might begin at the boundary of the simulation.

The dynamics of the ligand–protein contacts shows that relatively strong interactions with SER108/217 in chain A are maintained throughout the simulation. In chain B, an even stronger but variable interaction with SER108 is observed. At the same time, the interaction with SER217 is weak, and it disappears during the simulation time. In addition, the interaction of compound **1** with PRO105 in chain B is more stable than that with the identical amino acid in chain A (data can be found in Supplementary Material Figure S1).

The results of molecular docking and dynamics suggest that compound **1** can affect the AMPA receptor as a positive allosteric modulator. The values characterizing its affinity to the binding site are comparable to those of reference compounds (ampakines). The receptor binding mechanisms of action of compound **1** are also indirectly supported by a low BBB permeability along with an improved cognitive and locomotor activity in MCAO animals. Further investigation of specific receptors is required.

## 3. Materials and Methods

### 3.1. Chemistry

The purities of compounds for biological testing were assessed by NMR to be ≥95%. NMR spectra were detected on a Bruker Avance II 300 spectrometer, Germany (300 (^1^H), 75 MHz (^13^C)) in CDCl_3_ and DMSO-d_6_ in the pulse mode followed by Fourier transform, where Me_4_Si was used as an internal standard. Spin multiplicities were described as s (singlet), d (doublet), t (triplet), or q (quartet). IR spectra in the solid phase were recorded on a Bruker Tensor-27 instrument with the attenuated total internal reflectance (ATR) module. Refraction parameters were measured with an IRF-454B2M refractometer. Melting points were determined on a Stuart SMP10 instrument. Elemental analyses were carried out at the Laboratory of Organic Microanalysis of INEOS RAS. All initial reagents and solvents were purchased from Acros and Sigma-Aldrich.

*Ethyl 2-(2-Oxo-4-phenylpyrrolidine-1-yl)acetate (**3**):* A total of 20.84 g (0.521 mol) of sodium hydride (60% dispersion in mineral oil) was added in portions to 250 mL of DMSO. The reaction mixture was heated to 65 °C and stirred for 2.5 h. After cooling to 5 °C, a solution of 73.68 g (0.434 mol) of 4-phenylpyrrolidine-2-one in 200 mL of DMSO was added, followed by 94 mL (108.59 g, 0.868 mol) of ethyl chloroacetate. The mixture was stirred for 18 h at ambient temperature, then partitioned in a mixture of water and ethyl acetate. The organic layer was separated, the solvent was removed by evaporation (156–160 °C, 1.5 mm Hg), and the residue was purified by column chromatography (silica gel, ethyl acetate–toluene, 1:1) to afford 105.0 g (98%) of ester **3**. R_f_ = 0.3 (toluene/AcOEt 1:1). n_D_^20^ = 1.5355. ^1^H NMR (CDCl_3_, δ, ppm, J/Hz): 1.28 (t, J = 7.1, 3H, CH_3_CH_2_), 2.64–2.88 (m, 2H, H-3), 3.59–3.90 (m, 2H, H-5), 3.69–3.88 (m, 1H, H-4), 4.14, 4.23 (dd, J = 17, 7.2, 2H, NCH_2_C(O)), 4.28 (q, J = 7.1, 2H, OCH_2_CH_3_), 7.31–7.44 (m, 5H, Ph). ^13^C NMR (CDCl_3_, δ, ppm): 14.2 (CH_3_CH_2_), 37.3 (C-4), 38.5 (C-3), 44.1 (C-5), 55.0 (NCH_2_C(O)), 61.5 (CH_3_CH_2_), 126.9 (C_para_), 127.2 (2C, C_orto_), 128.9 (2C, C_meta_), 142.2 (C_ipso_), 168.5(NCH_2_C(O)), 174.9 (C-2). IR (ν/cm^−1^): 761, 1021, 1190, 1687 (C=O, lactam), 1740 (C=O, ester). Found (%): C, 67.87; H, 6.58; N, 5.28. Calcd. (%): C, 68.00; H, 6.93; N 5.66.

*2-(2-Oxo-4-phenylpyrrolidin-1-yl)acetic acid (**4**):* A mixture of 200 mL of propan-2-ol, 100 mL of water, and 47.61 g (0.730 mol) of potassium hydroxide was added to 105 g (0.424 mol) of ester **3**. The reaction mixture was stirred at 25 °C for 18 h, then poured into water, and 5 mL of concentrated hydrochloric acid was added. The product was extracted with ethyl acetate. The solvent was evaporated to afford 81.0 g (87%) of acid **4**. M.p. 161−163 °C. ^1^H NMR (DMSO-d_6_, δ, ppm, J/Hz): 2.42–2.72 (m, 2H, H-3), 3.58–3.79 (m, 2H, H-5), 3.23–3.61 (m, 1H, H-4), 3.94, 4.02 (dd, J = 17.1, 7.2, 2H, NCH_2_C(O)), 7.28–7.34 (m, 5H, Ph). ^13^C NMR (DMSO-d_6_, δ, ppm): 37.0 (C-4), 38.5 (C-3), 44.0 (C-5), 54.6 (NCH_2_CO), 126.9 (C_para_), 127.2 (2C, C_orto_), 129.1 (2C, C_meta_), 143.3 (C_ipso_), 170.7 (NCH_2_C(O)), 173.8 (C-2). IR (ν/cm^−1^): 765, 1237, 1272, 1623 (C=O, lactam), 1719 (C=O, ester). Found (%): C, 65.68; H, 5.74; N, 6.07. Calcd. (%): C, 65.74; H, 5.98; N, 6.39.

*2,5-Dioxopyrrolidin-1-yl 2-(2-oxo-4-phenylpyrrolidin-1-yl)acetate (**5**):* A total of 41 mL (29.77 g, 0.294 mol) of triethylamine, and 47.68 g (0.406 mol) of *N*-hydroxysuccinimide were added with stirring to a solution of 81.0 g (0.370 mol) of acid **4** in 250 mL of chloroform. After cooling to 5 °C, 51 mL (0.406 mol) of *N,N’*-diisopropylcarbodiimide was added. After 18 h, the precipitate formed was filtered off, washed with water, and concentrated with hydrochloric acid. The filtrate was washed with water and the organic layer was separated and evaporated to dryness to afford 110.0 g (94%) of ester **5**. R_f_ = 0.4 (toluene/AcOEt 1:1). ^1^H NMR (CDCl_3_, δ, ppm, J/Hz): 2.64–2.87 (m, 2H, H-3), 2.72 (s, 4H, -CH_2_CH_2_-, succin), 3.88–3.93 (m, 2H, H-5), 3.47–3.81 (m, 1H, H-4), 4.10, 4.21 (dd, J = 17.0, 7.2, 2H, NCH_2_C(O)), 7.29–7.37 (m, 5H, Ph). ^13^C NMR (CDCl_3_, δ, ppm): 25.4, 25.6 (-CH_2_CH_2_-, succin), 37.3 (C-4), 38.5 (C-3), 44.2 (C-5), 55.3 (NCH_2_C(O)), 126.8 (C_para_), 127.2 (2C, C_orto_), 128.9 (2C, C_meta_), 141.8 (C_ipso_), 171.3 (C(O), succin) 172.7 (C-2), 175.9 (NCH_2_C(O)). IR (ν/cm^−1^): 764, 1078, 1211, 1621 (C=O, lactam), 1698 (C=O). Found (%): C, 59.85; H, 4.89; N, 8.48. Calcd. (%): C, 60.75; H, 5.10; N, 8.86.

*Carbethoxymethyl ester of 2-oxo-(4-phenylpyrrolidino) acetic acid (**6**)*: A mixture of 80.8 g of 4-phenyl-2-pyrrolidone (**2**), 70 g of granular potassium hydroxide, and 500 mL DMSO was stirred at 90–100 °C for 2 h. Then, 152 g of ethyl chloroacetate was added dropwise to the hot reaction mixture. After 24 h, 550 mL of water was added to the reaction mixture and the mixture was extracted three times with 200 mL of chloroform. Fractionation (220–223 °C, 1.5 mm Hg) of extracts yielded 79 g (52%) of compound **6**, n_D_20 1.5215. ^1^H NMR (CDCl_3_, δ, ppm, J/Hz): 1.27 (t, J = 7.1, 3H, CH_3_CH_2_,), 2.40–2.75 (m, 2H, H-3), 3.12–3.39 (m, 1H, H-4), 3.56–3.81 (m, 2H, H-5), 4.16, 4.25 (dd, J = 17.0, 7.5, 2H, NCH_2_C(O)), 4.21, 5.04 (dd, J = 14.5, 7.1, 2H, OCH_2_C(O)), 4.26 (q, J = 7.1, 2H, OCH_2_CH_3_), 7.19–7.28 (m, 5H, Ph). ^13^C NMR (CDCl_3_, δ, ppm): 14.1 (CH_3_CH_2_O), 30.0 (C-4), 40.8 (C-3), 48.9 (C-5), 50.4 (NCH_2_C(O)), 60.1 (OCH_2_C(O)), 61.5 (OCH_2_CH_3_), 125.9 (C_para_), 126.1 (2C, C_orto_), 128.7 (2C, C_meta_), 148.1 (C_ipso_), 169.2, 169.5 (2C, C(O)), 173.7 (C-2). IR (ν/cm^−1^): 761, 1021, 1190, 1687 (C=O, lactam), 1740 (C=O, ester). Found (%): C, 69.47; H, 6.58; N, 4.32. Calcd. (%): C, 69.94; H, 6.27; N 4.59.

*Potassium 2-[2-(2-oxo-4-phenylpyrrolidin-1-yl)acetamido]ethane-1-sulfonate (**1**):* Sche me 2 (**2**→**6**→**1**, method 1). A mixture of 6 g (0.02 mol) of carbethoxymethyl ester of 2-oxo-4-phenylpyrrolidino) acetic acid (**6**) in 3 mL of DMF was stirred with 6.52 g (0.04 mol) of potassium taurate in 5 mL of water until the starting ester disappeared in the mixture (TLC (thin layer chromatography), silufol, ethyl acetate) for 3 days. Then, the formed crystals were removed by filtration, the mother liquor was evaporated, and 10 mL of ethanol was added to the residue. The mixture was stirred until a homogeneous mass was formed, to which, 10 mL of acetone and 20 mL of ether were added. The precipitate was filtered off, washed with acetone, and then washed with 10mL each of acetonitrile and ether. After drying the crystals in a desiccator, 9.85 g (90%) of sulfonate was obtained in compound **1**.

Scheme 2 (**2**→**3**→**4**→**5**→**1**, method 2). A solution of 44.41 g (0.348 mol) of 2-aminoethanesulfonic acid and 19.51 g (0.348 mol) of potassium hydroxide in 200 mL of water were added to a solution of 81.0 g (0.370 mol) of acid **4** in 250 mL of chloroform. The mixture was stirred for 15 min, then another 200 mL of water was added, followed by stirring for a further 18 h. The reaction mixture was evaporated and the residue was stirred with 150 mL of propan-2-ol and evaporated again to dryness. The last step was repeated twice. The residue was suspended in 800 mL of acetonitrile and 50 mL of ethyl acetate. After 18 h, the supernatant was decanted, and the precipitate was washed with acetonitrile. The remaining solvents were evaporated to afford 119.9 g (94%) of the compound **1**. ^1^H NMR (DMSO-d_6_, δ, ppm, J/Hz): 2.43–2.68 (m, 2H, H-3), 2.56 (t, J = 6.9, 2H, NHCH_2_CH_2_SO_3_), 3.31–3.39 (m, 2H, NHCH_2_CH_2_SO_3_), 3.21–3.57 (m, 2H, H-5), 3.64 (m, 1H, H-4), 3.74–3.80 (m, 2H, H-5), 3.80, 3.86 (dd, J = 16.4, 5.6, 2H, NCH_2_C(O)), 7.23–7.38 (m, 5H, Ph), 7.96 (br. s, 1H, NH). ^13^C NMR (DMSO-d_6_, δ, ppm): 35.9 (NHCH_2_CH_2_SO_3_), 37.2 (NCH_2_C(O)) 37.2 (C-4), 38.5 (C-3), 50.6 (C-5), 54.7 (NHCH_2_CH_2_SO_3_), 127.1 (2C, C_orto_), 127.5 (C_para_), 129.0 (2C, C_meta_), 143.1 (C_ipso_), 167.5 (C(O)), 173.9 (C-2). IR (ν/cm^−1^): 739, 1042, 1190, 1665 (C=O). Found (%): C, 45.98; H, 4.61; N, 7.37; S 9.03. Calcd. (%): C, 46.14; H, 4.70; N, 7.69; S 8.80.

### 3.2. In Silico Studies

#### 3.2.1. The Structure–Activity Relationship (SAR)

Biological activity and molecular mechanisms of action were predicted by the web service PASS (Prediction of Activity Spectra for Substances) Online [8]. The results of the prediction are presented as a list of activities with possibilities: Pa is the possibility that the compound belongs to the class of compounds exhibiting the activity; Pi is the possibility that the compound does not belong to the class of compounds exhibiting the activity. The acute rat toxicity (LD_50_) was estimated using the web service GUSAR Online Acute Toxicity: http://www.way2drug.com/gusar/acutoxpredict.html accessed on 19 August 2021 [36]. This service includes QSAR models predicting LD_50_ values for p.o., i.p., i.v., and s.c. routes of administrations. The QSAR models were created using the SYMYX MDL Toxicity Database. The combination of QNA (Quantitative Neighborhoods of Atoms) descriptors, PASS prediction results, and a self-consistent regression were used for the development of QSAR models. RMSE values of QSAR models for the test sets varied from 0.57 to 0.68 Log10(LD_50_ (mmol kg^−1^)). The detailed information related to the accuracy of QSAR models is represented in the publication and the website of the service.

#### 3.2.2. Calculation Details

The affinity of the studied ligands for the ligand-binding domain of the AMPA receptor was assessed using molecular modeling methods as previously described [36,37,38,39]. All calculations were performed using Schrodinger Suite software [40].

The calculations are based on a physical model with the assumption that the permeability is dominated by the free energy of the desolvation and change in state (neutralization and tautomerization) on passing into the membrane. The membrane is modeled as a low-dielectric continuum, and water as a high-dielectric continuum [41].

#### 3.2.3. Protein and Ligand Preparation

The geometric parameters of the ligand-binding domain of the AMPA receptor in a complex with aniracetam (PDB code 2AL5) were downloaded from the Protein Data Bank [35,42]. Model protein structures were prepared using the Schrodinger Protein Preparation Wizard tool: hydrogen atoms were added and minimized; missing amino acid side chains were added; bond multiplicities were restored; solvent molecules were removed; the entire structure was restrained and optimized in the OPLS3e force field at the physiological pH value [43].

The geometric parameters of the potential PAM of the AMPA receptor were also optimized, taking into account all permissible conformations.

#### 3.2.4. Active Site Analysis

The PAM binding site is located in a U-shaped cleft, filled with solvent, at the boundary of two polypeptide chains. The cleft, formed by the PRO105–SER108 sequence, is the key element of the binding site [35]. The functional amino acids are SER108, PRO105, SER217, and ASN242 (PDB code 2AL5), which can form water-mediated interactions with the ligand. The re-docking of the native ligand correctly reproduces the actual aniracetam position obtained from crystallographic data, with an RMSD value of 0.366 A (data can be found in Supplementary Material Figure S2).

#### 3.2.5. Molecular Docking and Dynamics

Docking was performed using the induced fit docking method (IFD) with standard prediction accuracy. The docking grid matrix was selected according to the ligand size. Amino acids within a radius of 5 Å were optimized to a limited extent with reference to the native ligand. This docking protocol took into account the influence of the native ligand on the protein conformation. The key values (glide score, ligand efficiency, and IFD score) obtained by the docking were compared with the corresponding values for aniracetam, which was used as a reference structure. The binding energy parameters of piracetam were also evaluated because piracetam was used as a reference compound for biological tests. In addition, the binding energy components of the resulting ligand–protein complexes were calculated for several other ligands using the generalized dielectric Born model (MM-GBSA). In all cases, water was used as the solvent. The data obtained by molecular docking were used for subsequent molecular dynamics simulations. The ligand–protein complex was placed in an orthorhombic system with a buffer zone 26 Å from the protein surface. The system was filled with a 0.15 M aqueous solution of NaCl. The solvent model TIP3P was used. The resulting system was minimized and equilibrated using a standard internal protocol (8000-step minimization with steepest descent method, four steps of restrained minimization: solvent, solute, common at 0 K last step—equilibration at 310 K). The main period of molecular dynamics simulation was 100 nanoseconds at a temperature of 310 K (body temperature). The environment was NVT, with a recording interval of 10 ps. All calculations were performed using Desmond software included in the Schrodinger suite.

#### 3.2.6. Calculation Method of BBB Permeability

Description of ADME descriptors: QPPCaco predicted the apparent Caco-2 cell permeability in nm s^–1^. Caco-2 cells are a model for the gut–blood barrier. QikProp predictions are for non-active transport. (<25 poor, >500 great). QPPMDCK predicted the apparent MDCK cell permeability in nm s^–1^. MDCK cells are considered to be a good mimic model for the blood–brain barrier. QikProp predictions are for non-active transport (<25 poor, >500 great). QPlogBB predicted the brain/blood partition coefficient. Note: QikProp predictions are for orally delivered drugs, so, for example, dopamine and serotonin are CNS-negative because they are too polar to cross the blood–brain barrier (−3.0–1.2).

The membrane permeability tool allows for the calculating of the passive membrane permeability of a set of molecules. It is primarily intended for use on a congeneric series of ligands to evaluate the relative permeability of similar ligands. The calculations are based on a physical model, with the assumptions that the permeability is dominated by the free energy of the desolvation and change in state (neutralization and tautomerization) on passing into the membrane. The membrane is modeled as a low-dielectric continuum, and water as a high-dielectric continuum. A conformational search is performed in the low-dielectric continuum and the low-energy conformers are evaluated and scored in the high-dielectric continuum. Macrocycles are handled with a specialized sampling method. The highest value of the permeability found from this ensemble is used as the permeability for that molecule. In addition to this approach, another approach is used, in which the model is optimized to reproduce RRCK permeability assay results, with fitted energy and volume terms.

### 3.3. In Vitro Studies

The primary culture of newborn rat cortical neurons was obtained as previously described [44]. Briefly, cortical neurons were collected from newborn Wistar rats and inoculated onto culture plates (4 × 10^5^ cell cm^–2^). The cells were cultured in a medium containing 92.75% NeurobasalTM medium (Invitrogen), 5% fetal calf serum (PanEko), 2% B27 supplement (Invitrogen), and 0.25% L-glutamine (Invitrogen) at 37 °C under a humidified atmosphere containing 5% CO_2_. We added ARAC after 24 h and did not wash it; thus, cells were in the culture with ARAC for 24 h in total. Then, we reduced the ARAC concentration by diluting it: we replaced 1/3 of the medium every 2 days, for a total of four changes. Cells grown in the tissue culture plate for 10 days were photographed using a Zeiss Observer inverted phase contrast microscope.

After cultivation for 10 days, the medium was replaced with saline (145 mM NaCl, 2.5 mM KCl, 10 mM *N*-(2-hydroxyethyl)piperazine-*N*-(2-ethanesulfonic)acid, 10 mM glucose, 2 mM CaCl_2_, 1 mM MgCl_2_ and 2 μM glycine; pH 7.3). Glutamate (50 μM) was added to the buffer for 15 min. Then, the cells were washed with saline. Then, the cells were placed into the medium without B27 and treated with compound **1** at concentrations of 10, 50, and 100 μM (all diluted in 50 μL) or 50 μL saline. The total volume of the culture was 500 μL. After 3 h of incubation, the culture was washed with saline and incubated for 24 h in the medium without B27. B27 was excluded from the culture to eliminate its neuroprotective features. Finally, an MTT assay of the cell viability was performed.

The cell viability was measured according to the cell ability of tetrazolium dye reduction. The MTT solution was added to the culture medium at a final concentration of 0.5 mg mL^−1^, and the cells were left in darkness for 4 h at 37 °C. After that, the solution was removed, and formazan crystals were dissolved in dimethyl sulfoxide. The formazan absorption at 492 nm was measured using a Zenyth 1100 (Anthos Labtec Instruments, GmbH, Salzburg, Austria) microplate reader.

### 3.4. Analysis of Compound **1** in Rat’s Plasma and Brain by HPLC-MS/MS

#### 3.4.1. Preparation of Working Solutions

Stock solutions were prepared by dissolving a weighed portion of a substance of known concentration in deionized water to obtain a concentration of 1 mg mL^−1^.

Brain. A brain sample weighing 50 mg (± 2 mg) was placed in 2 mL tubes and 10 μL of an aqueous solution of an internal standard with a concentration of 1 μg mL^−1^ was added, then 140 μL of a 1% aqueous solution of formic acid was added and homogenized. Brain samples were ground in a Precellys Evolution homogenizer using 2 mL tubes with ceramic balls at room temperature at a speed of 4500 vibrations min^−1^ for 2 cycles of 90 s. After that, 400 μL of chloroform was added. The sample was shaken and centrifuged at 10,621 g for 2 min. The aqueous layer of the liquid was taken and 400 μL of chloroform was added. The sample was shaken and centrifuged at 10,621 g for 2 min. The supernatant was taken for analysis.

Plasma. A total of 10 μL of an aqueous solution of an internal standard with a concentration of 1 mg mL^−1^ was added to 50 μL of plasma. A total of10 μL of formic acid and 150 μL of a 0.2% solution of formic acid in methanol was added to the solution. A total of 780 μL of deionized water was added to the resulting solution. The solution was centrifuged at 10,621 g for 2 min. The supernatant was collected and analyzed.

#### 3.4.2. HPLC-MS Conditions

Analyses were performed by a Shimadzu 20 (Shimadzu, Kyoto, Japan) system consisting of HPLC coupled to triple quadrupole mass spectrometer Shimadzu 8040 (Shimadzu, Japan). The chromatographic separation was performed on Discovery C18 column 3 × 150 mm, 5 μm in a gradient mode with mobile phase components A (0.01M ammonium acetate in water) and B (acetonitrile). The gradient cycle was performed in the following way: 0–6 min 5–40% B, 6.01–10 min 40–95%, 10.01–15 min, isocratic elution with 100% B, and then returned to the initial condition. The column temperature was 60 °C. The sample injection volume was 20 μL. The autosampler temperature was 8 °C.

The electrospray ionization (ESI) source was set in positive ionization mode. Multiple reaction monitoring was used to perform mass spectrometric quantification. MS conditions: interface voltage 3,500 V (ESI−), nebulizer gas (nitrogen) flow 2.5 l min^−1^, drying gas (nitrogen) flow 15  L min^−1^, CID gas pressure 230 kPa, DL temperature 200 °C, and heat block temperature 500 °C. High-purity argon was used as collision gas. The precursor and product ions (m/z) of target analytes were 327.10 and 174.10 for compound **1** and 236.10 and 154.15 for internal standard; collision energy was −20 V for both compounds.

Total information data can be found in Supplementary Material Figures S3 and S4.

### 3.5. Animals

Fifty specific pathogen-free 3 months old, male Wistar rats were obtained from the Pushchino Nursery for Laboratory Animals (Branch of the Institute of Bioorganic Chemistry, Russian Academy of Sciences, Moscow, Russia) and maintained according to the standards of the 2010/63/EU Guidelines on the treatment of animals used for research purposes. The animals were kept in a conventional animal facility of the Pirogov Russian National Research Medical University under an automated day (08:00 to 20:00) and night (20:00 to 08:00) cycle with at least 12-fold exchange of air per hour and optimal temperature and humidity of 20–24 °C and 45–65%, respectively.

The experimental protocols were approved by the Pirogov Russian National Research Medical University Animal Care and Use Commission (application No. 48/2018). The animal procedures were of medium severity and caused short-term medium-level pain or stress. The rats were euthanized using a CO_2_ chamber.

Before conducting the experiments, the animals underwent the HBT and were allocated to groups based on their behavior score and weight. Kullback–Leibler divergence method for allocation based on two numerical parameters was used for allocation. We used a sum score for behavior tests and weight. Subsequently, two experimental series were performed. In the first series, rat behavior without stroke modeling after injection of compound **1** (experimental group, *n* = 10) or saline (control group, *n* = 10) was evaluated (Scheme 3a). In the second series, compound **1** (*n* = 10), saline (*n* = 10), and piracetam (*n* = 10) were intravenously injected at 24 h after establishing the stroke model and subsequently daily for the next 5 days. Compound **1** was injected at a dose of 125 mg kg^−1^ and an injection volume of 0.5 mL. Piracetam (buffer solution for intravenous and intramuscular injection 20%) was injected at a dose of 300 mg kg^−1^. For the experiments, the following predetermined exclusion criteria were used: (1) incomplete MCAO indicated by an incomplete lesion on magnetic resonance imaging (MRI) after 24 h [*n* = 4]and (2) post-operative weight loss of > 20% and severe animal condition within the first post-stroke 48 h [*n* = 3]. Animals reached humane endpoints based on the absence of nociceptive reflex withdrawal responses, hypothermia, and heart rate reduction to 100–150 beats min^−1^. As steroid treatment could have influenced the experiment, rats were humanely euthanized according to the ethical protocol. The experiment design is presented in Scheme 3b.

#### 3.5.1. Transient Middle Cerebral Artery Occlusion

The rats were anesthetized initially using 3% isoflurane and maintained with a mixture of 2–2.5% isoflurane and 97.5–98% atmospheric air (EZ-7000 Classic System, E-Z Anesthesia^®^ Systems). 0.05 mg kg^−1^ atropine sulfate dissolved in 1 mL 0.9% NaCl was injected intraperitoneally to reduce respiratory tract secretion. In addition, 0.1 mL of 0.5% ropivacaine was injected subcutaneously along the prospective incision site (neck midline). Subsequently, a transient MCAO was conducted for 90 min according to the protocol proposed by Koizumi and modified by Longa. This was assisted by MRI, which allowed for simultaneous visualization of previously described [45].

#### 3.5.2. MRI Measurements

During the procedure, the rats were maintained under inhalation anesthesia as mentioned above. MRI measurements were obtained intraoperatively as previously described and on postoperative days 1, 7, 14, and 28 after operation using a ClinScan (Bruker BioSpin, Billerica, MA, USA) 7T MRI system. We performed T2-weighted imaging to evaluate ischemic lesions (Turbo Spin Echo pulse sequence with restore magnetization pulse and breath synchronization, turbo factor 10, repetition time/echo time 5230/46 ms, averages 2, spectral fat saturation, a field of view 30 × 21.1 mm, slice thickness 0.7 mm, matrix size 256 × 162). MRI data analysis was performed using ImageJ software [46]. The volume of the hyperintensity ischemic stroke area was calculated by summing the areas of adjacent cross-sections using the following formula: Volume = (S1 + … + Sn) × (h + d), where S1, …, Sn are areas measured in n slices, h is the slice thickness, and d is the interval between slices.

#### 3.5.3. Behavioral Tests

**Figure 8:**
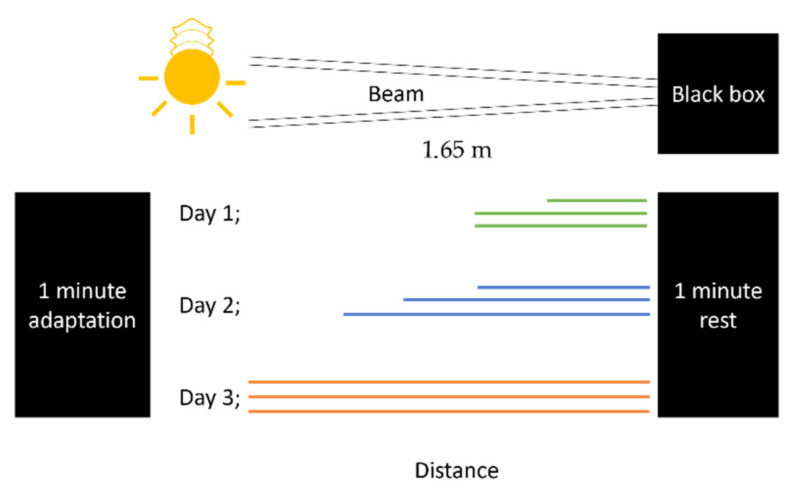
Beam walking test. Animals were trained three times per day during 3 days before MCAO and on days 12–14 after MCAO. First, animals are placed in the black box with a nesting material from the cage for 1 min to make them comfortable during the test. Then, animals are placed in different distances from the black box and are trained to reach the black box. Once they succeed, they rest for 1 min in the box. On day 14 after MCAO, the test with whole distance is repeated, and the number of steps, errors, and slides are counted. Each line represents one attempt. Colors correspond to different days of training.

In the first experimental series, the following test systems were used: HBT (OpenScience, Russia), OFT (OpenScience), and LDT (OpenScience) [11]. The design for the second experimental series was as follows. All rat behavioral changes were evaluated during the four weeks after stroke simulation. Individuals who performed these procedures were double-blinded. General neurological disorders were assessed using the modified neurological severity score (mNSS) [12]. Motor and cognitive impairment were evaluated using the following functional tests: BWT (OpenScience), HBT (OpenScience), and OFT (OpenScience). The BWT was performed between postoperative days 12 and 14. In this test, motor dysfunction in all four limbs was evaluated; specifically, the number of steps, errors, and slides were counted. The overall score was calculated using the following formula: score = errors + 0.5 × slides × 100/total number of steps [47]. The beam apparatus consists of 165 cm beams with a flat surface of 10 mm or 6 mm width, resting 100 cm above the table top on two poles. A black box is placed at the end of the beam as the finish point. Nesting material from home cages is placed in the black box to attract the rat to the finish point. A lamp (with 60-watt light bulb) is used at the start to stimulate the animals to move towards the end. The training pipeline is presented in Figure 8 [48,49].

The HBT was performed on postoperative days 10 and 24. In this test, the following parameters were assessed: orientational and motor activity (horizontal activity, arbitrary units: number of crossings; vertical activity, arbitrary units: number of head dips), anxiety level (immobility and grooming time and number of grooming and immobility events), and the number of central sector crossings. The OFT was performed on postoperative days 16 and 26. The parameters measured in this test were similar to those used to assess orientational and motor activity. In addition, the total walk length and movement rate were calculated using the Ethovision 12 software.

### 3.6. Statistical Analysis

All statistical analyses were performed using the IBM^®^ SPSS^®^ Statistics Version 23.0 and R software Version 3.3.2 (R Foundation for Statistical Computing, Vienna, Austria). For normally distributed quantitative continuous variables, the mean and standard deviation (SD) values were calculated and presented as descriptive statistics. For ordinal variables, median and 25- and 75-percentiles were calculated. For qualitative variables, the absolute count and percentage value for each category were calculated. For quantitative variables, the normality of distribution was verified using frequency histograms and Kolmogorov–Smirnov test. A two-tailed *p*-value < 0.05 was considered statistically significant. To compare changes in structure displacement, lesion size, and neurological scale, a general linear repeated measures model with a group as a fixed factor was used. One-way multivariate analysis of variance was used in the analysis of behavior. Experimental groups were designed to have an equal sample size (8 vs. 8 rats) based on sample size calculations for the independent sample t-test using Statistica 10 software (type I error 0.05, type II error 0.20). Animal survival was estimated by the Kaplan–Meier method using the log-rank test.

## 4. Conclusions

Cognitive and memory dysfunction after ischemic stroke have a huge impact on a patient’s quality of life, and new methods are in high demand. Here, for the first time, we describe potassium 2-[2-(2-oxo-4-phenylpyrrolidin-1-yl)acetamido]ethane-1-sulfonate (**1)**, a new racetam family member, as a potential agent for ischemic stroke management. The evaluation of the biological activity of compound **1** using the PASS Online service demonstrated the potential anti-ischemic effect of this substance in an in vitro glutamate toxicity experiment, where compound **1** showed a high neuroprotective efficacy, which allowed us to continue the investigation.

According to the computer simulation data, as well as in vivo studies in intact animals, compound **1** undergoes almost no passive diffusion through the BBB (compound **1** concentration in the brain is 0.3 μg g^–1^). However, even a low concentration was sufficient for statistically significant changes in the animal behavior. This may indicate active receptor interactions of compound **1**. The structural similarity of compound **1** to such drugs as piracetam, aniracetam, and CMPDA suggests an interaction with the AMP receptor (AMPAR allosteric modulator). This conclusion is supported by a computer simulation, which showed that compound **1** occupies the U-shaped gap of the S1S2 binding site, forms several close bonds with functional amino acids, and remains in the allosteric site for at least 100 ns without losing the bonds.

On the one hand, compound **1** has non-significant effects on the survival rate of animals after MCAO. In addition, it does not affect the rate of the cerebral infarct volume reduction according to multiple control MRI tests. On the other hand, compound **1** improved functional and cognitive recovery and was associated with better learning capabilities. This improvement may be due to the effects of compound **1** on viable brain tissue and the stimulation of neuroplasticity.

Thus, the improvement of cognitive function and the similarity of the chemical structure of compound **1** with that of piracetam and aniracetam, as well as computer simulation data (Table 7), indicate that compound **1** may be an AMPAR allosteric modulator. Although the direct mechanism of action of compound **1** is unclear, we believe that our in vivo data indicate the necessity of further investigation of compound **1** as a powerful modulator of neuronal activity.

In the postischemic stroke model, compound **1** showed a significant improvement in both the anxiety level and orientational activity. Thus, compound **1** may become a new option for patients’ rehabilitation after stroke. However, we believe that compound **1** may also improve other neurodegenerative conditions, such as those associated with head traumas, aging, and age-related pathologies and seizures. In addition, it may be beneficial for patients with depression and cognitive deficits after COVID-19 infection, as this condition can persist for a long period and significantly affect the patient’s wellbeing.

In summary, compound **1** had high neuroprotective properties, improved the number of sections crossed during different cognitive and behavioral tests, such as the open field test, hole-board test, and others. In addition, the substance significantly reduced the immobility time for rats after MCAO. Notably, it had an effect on the learning capabilities of rats, as only compound **1**-receiving rats were able to complete the beam walking test. Therefore, this data suggest that compound **1** is highly effective and further investigation of the mechanism of action and the effects in other conditions is needed.

## Figures and Tables

**Table 1:** PASS Online prediction results for compound **1** regarding nootropic and cerebral anti-ischemic effects.

Effect	Pa	Pi	Mechanism of Action	Pa	Pi
Nootropic	0.705	0.040	Neurotransmitter antagonist	0.502	0.039
Antianginal	0.544	0.032			
Antihypoxic	0.553	0.025			
Cytoprotective	0.609	0.027			

Pa—the probability that the compound belongs to the active compound class for this activity. Pi—the probability that the compound does not belong to the active compound class for this activity. The greater the difference between Pa and Pi, the greater the probability that this activity will be confirmed in experiments.

**Table 2:** Animal activity in the open field test (OFT).

	Compound 1	Normal Saline	Piracetam
Crossings (n)	53.7 ± 10.3	53.6 ± 14.2	65.4 ± 20.4
Number of center sector crossings (center) (n)	1.6 ± 0.6 *	0.8 ± 0.9	1.4 ± 1.2
Vertical activity (n)	10.3 ± 4.3 *	16.8 ± 6.1	15.9 ± 7.5
Immobility duration (sec)	12.8 ± 8.4*	25.6 ± 13.8	16.0 ± 10.4
Grooming time (sec)	40.6 ± 33.2	37.1 ± 21.6	14.4 ± 9.4

* *p* < 0.05 compared to the normal saline group. The results are presented as mean and SD.

**Table 3:** Animal behavior in the light–dark box test (LDB).

	Compound 1	Normal Saline	Piracetam
Time spent in the light compartment (sec)	16.5 ± 8.3 *	9.8 ± 5.2	26.3 ± 13.4 *
Number of crossings (n)	2.9 ± 1.2 *	1.4 ± 0.8	3.7 ± 1.6 *
Number of peeking out events (n)	5.2 ± 2.8	1.6 ± 1.4	4.8 ± 2.5

* *p* < 0.05 compared to the normal saline group. The results are presented as mean and SD.

**Table 4:** Mean beam walking test scores on day 14 after MCAO.

	Compound 1	Normal Saline	Piracetam
Left forelimb score	42.2 ± 28.0	26.0 ± 24.2	32.8 ± 26.4
Right forelimb score	11.6 ± 13.8	16.6 ± 17.2	12.4 ± 16.0
Left hindlimb score	28.8 ± 15.3	28.3 ± 30.2	24.8 ± 22.2
Right hindlimb score	45.8 ± 30.4	35.7 ± 32.6	36.0 ± 30.4

The results are expressed as mean ± SD.

**Table 5:** Predicted permeability of ampakines in silico and in the blood–brain barrier (BBB).

Ampakines	Log P_m_^1^	P_Caco_, cm s^−1^ (QPPCaco) ^2^	P_MDCK_, cm s^−1^ (QPPMDCK) ^3^	QPlogBB ^4^	Literature Report of BBB Permeability
Seletracetam	−4.4	279.0	1268.6	−0.4	Very good [19]
Oxiracetam	−4.7	56.0	82.73	−1.0	Low [20]
Piracetam	−4.5	165.0	261.5	−0.5	Very good [21,22]
Fasoracetam	−4.5	227.8	410.6	−0.4	Low (30%) [23]
Phenylpiracetam	−4.6	155.3	252.1	−0.7	Unknown
Compound 1	−6.5	20.5	20.7	−1.5	Low

^1^ Log Pm—the logarithm of the membrane permeability in cm s^–1^; ^2^ QPPCaco—predicted apparent Caco-2 cell permeability in nm s^–1^. Caco-2 cells are used as a model of the gut–blood barrier. QikProp predictions are for non-active transport. (<25—poor, >500—excellent); ^3^ QPPMDCK—predicted apparent MDCK cell permeability in nm s^–1^. MDCK cells are a preferable model of the blood–brain barrier. QikProp predictions are for non-active transport. (<25—poor, >500—excellent); ^4^ QplogBB—predicted brain/blood partition coefficient. QikProp predictions are available for oral drugs. For example, dopamine and serotonin are CNS-negative because they are too polar to cross the blood–brain barrier.

**Table 6:** The plasma concentration ratio of compound **1** in the striatum and cerebral cortex in intact animals and rats 24 h after MCAO.

Rats	C_plasma mkg_ _mL_^−1^	C_striatum_ _ng mg_^−1^	C_cortex_ _ng/mg_	C_plasma_/C_striatum_	C_plasma_/C_cortex_
Intact animals	16.1 ± 5.8	247.2 ± 42.6	255.1 ± 78.2	651.2 ± 52.0	630.9 ± 98.6
Animals after MCAO	13.8 ± 3.6	1533.6 ± 24.5	3633.8 ± 40.1	89.7 ± 9.4	37.9 ± 6.0

**Table 7:** Results of the docking procedure at the active site of the ligand-binding domain (S1S2) of the AMPA receptor.

ID Structure	Glide Score, kcal mol^–1^	|LE|	IFD Score, kcal mol^–1^	ΔG_MM-GBSA_, kcal mol^–1^	H-bond Bridges
Aniracetam	−10.17	0.63	−1114.25	−42.28	Water-SER-A-108; Water-SER-B-108
Piracetam	−8.83	0.88	−1115.15	−25.19	Water-SER-A-108 Water-ASN-B-242 Ser-A-217
CMPDA	−10.69	0.43	−1110.25	n/d	Pro-A-105 Pro-C-105
**1-*R***	−11.65	0.53	−1118.45	−34.42	SER-A-108
**1-*S***	−9.91	0.45	−1116.58	−19.48	Water-SER-A-217

|Ligand Efficiency|—the ratio of glide score to the number of heavy atoms. *n/d-data not detected*

## Data Availability

The data presented in this study are available in this article.

## Supplementary Materials

The following are available online, Figure S1: Visualization of S1S2 LBD of AMPA-receptor, Figure S2: Intensity of ligand–protein contacts in the system. Figure S3: Chromatograms of compound **1** with IS in brain homogenate, Figure S4: Chromatograms of compound **1** with IS in brain homogenate from animal with MCAO.

## Sample Availability

Samples of compounds are available from the authors.
