# Supplementary material for: A Novel Phenylpyrrolidine Derivative: Synthesis and Effect on Cognitive Functions in Rats with Experimental Ishemic Stroke
Source: Molecules. 2021 Oct 11;26(20):6124. doi: 10.3390/molecules26206124 (PMC8541353; doi:10.3390/molecules26206124)
Supplement: Supplementary file 1 [file molecules-26-06124-s001.zip › molecules-1369294-supplementary.pdf]

Supplementary Materials

GluR2 ligand-binding core (S1S2J) of AMPA-receptor was chosen as biological target. The crystal structure protein with aniracetam was downloaded from Protein Data Bank. Aniracetam redocking into the LBD-site correctly reproduced the mode of receptor and native ligand binding determined by X-ray crystallography (Figure A1). The root-mean-square deviation (RMSD) for ligand was 0.366

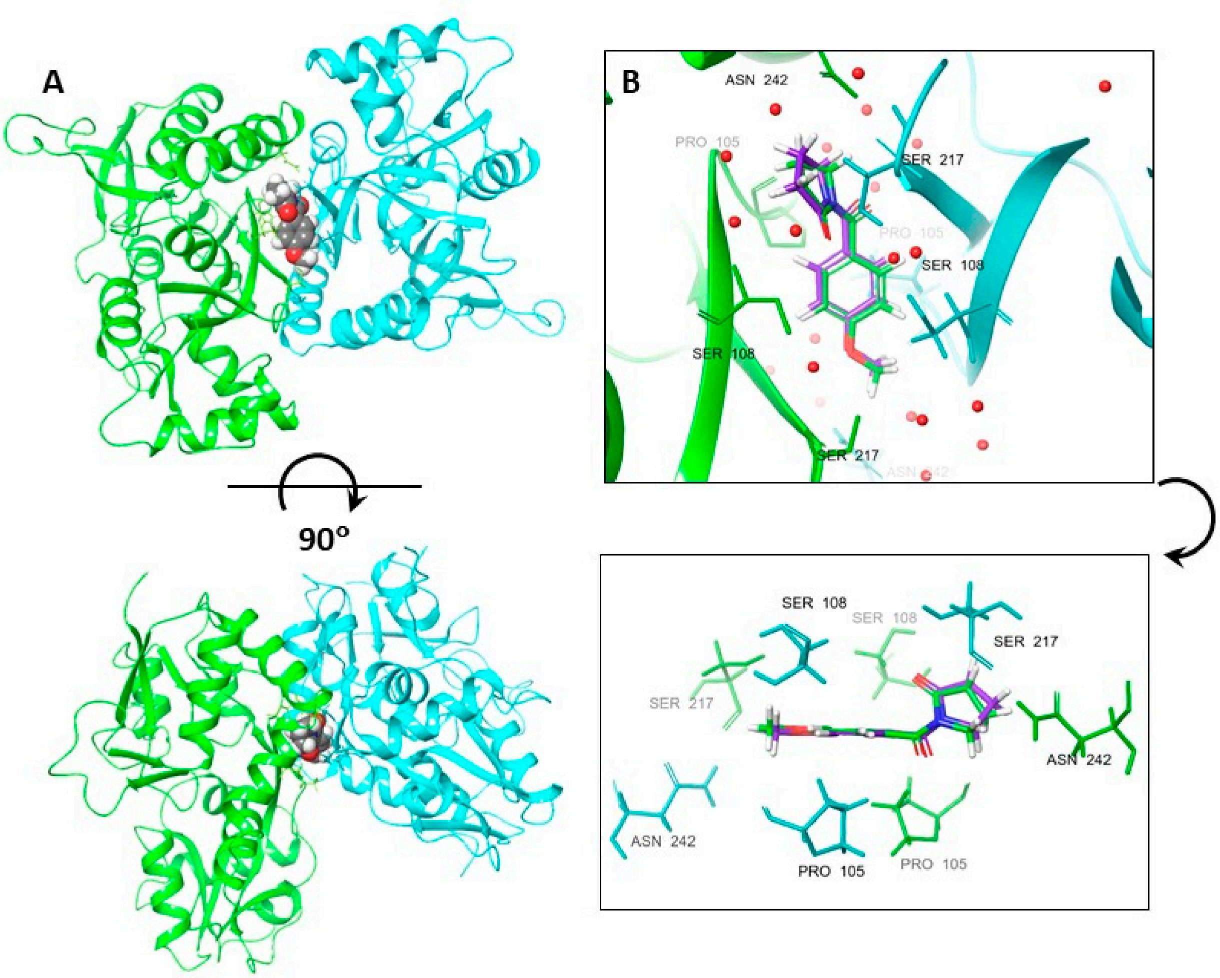

Figure-S1 Visualization of S1S2 LBD of AMPA-receptor: A – position of the aniracetam in the active site of the LBD (PDB code 2AL5); B – superimposition of two molecule of aniracetam: green molecule was downloaded from Protein Data Bank; violet was obtained as result of redocking procedure.

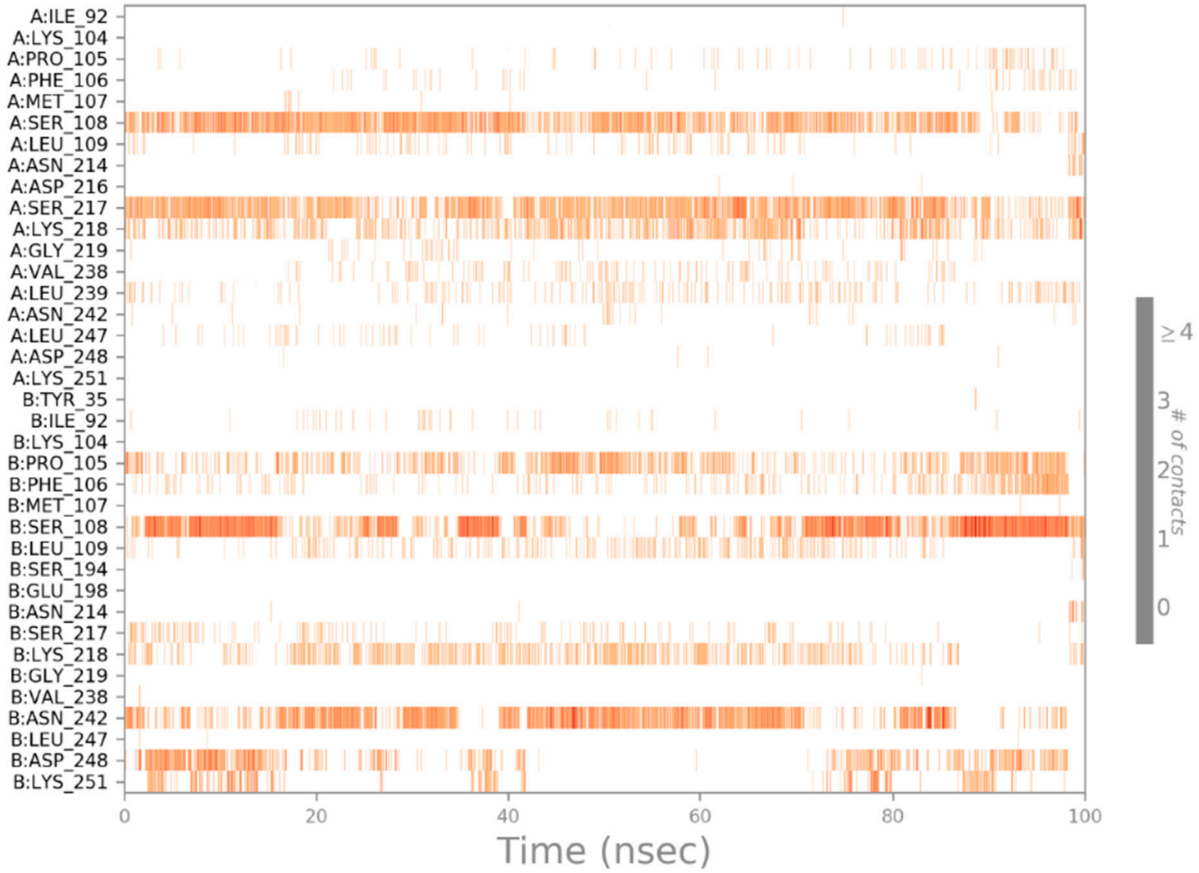

Figure-S2 Intensity of ligand-protein contacts in the system

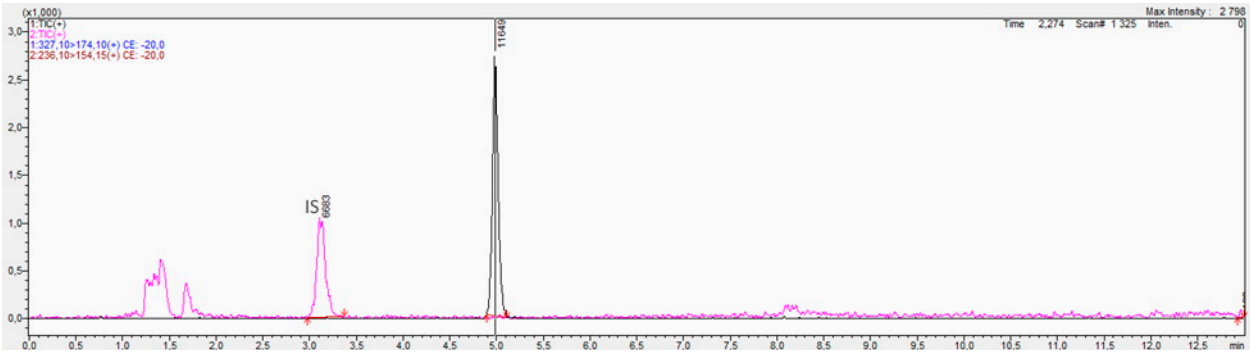

Figure-S3 Chromatograms of compound 1 with IS in brain homogenate

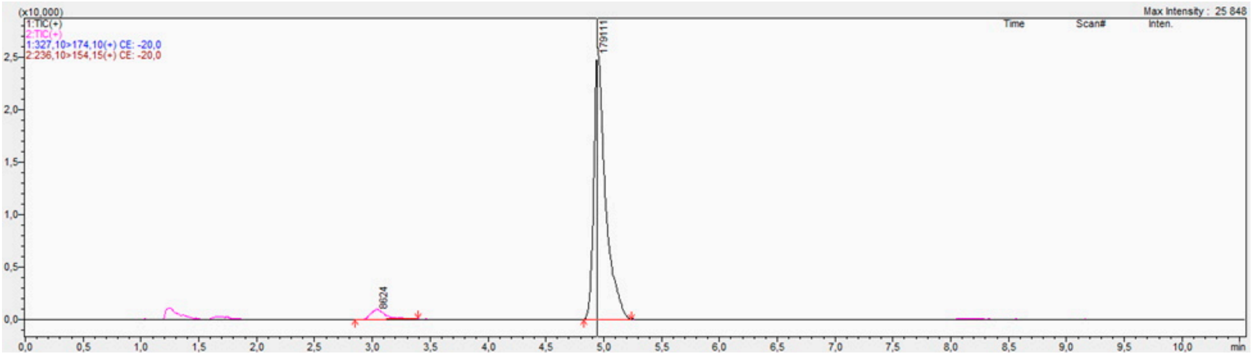

Figure-S4 Chromatograms of compound 1 with IS in brain homogenate from animal with MCAO.
